# Geo-spatial analysis of high-risk fertility behaviors and child stunting in Ethiopia

**DOI:** 10.3389/fpubh.2024.1449689

**Published:** 2024-12-18

**Authors:** Wondaya Fenta Zewdia, Daniel Asmelash, Yemane Asmelash

**Affiliations:** ^1^Department of Statistics, College of Science, Bahir Dar University, Bahir Dar, Ethiopia; ^2^College of Medicine and Health Sciences, Mizan-Tepi University, Mizan-Aman, Ethiopia; ^3^Department of Statistics, College of Science, Aksum University, Aksum, Ethiopia

**Keywords:** association, birth, high risk fertility behavior, spatial, spatial Durbin model, stunting, zones, Ethiopia

## Abstract

**Background:**

The process of childbirth involves significant risks, particularly when certain high-risk fertility behaviors (HRFBs) are observed. HRFB of birth includes maternal age below 18 years or above 34 years at the time of childbirth, having a child born after a short birth interval (24 months), and having a high parity (more than three children). The majority of child stunting cases were linked to high-risk reproductive practices. Stunted children are those whose height-for-age Z-score is less than –2SD (standard deviation) from the median of a reference population. This study aimed to investigate the spatial association between HRFB of birth and stunting in under-five children across Ethiopia’s administrative zones.

**Method:**

This study used cross-sectional data from the latest Mini Ethiopian Demographic and Health 2019 Survey, which included a weighted sample of 4,969 under-five children from 64 administrative zones. Spatial model analysis, specifically the spatial Durbin model, was used to examine the association between HRFB of birth and stunting in children. ArcGIS 10.8 was used for mapping and SAS 9.4 was used for model analysis.

**Results:**

The average mean proportion of HRFB of birth to the rate of stunting in children at the zonal level in Ethiopia was observed to be 58 and 36%, respectively, across Ethiopian zones. Children whose mothers displayed HRFB of birth and who were stunted were 24% at all times. The median value of HRFB of birth and stunting were 0.61 and 0.36, respectively. The average vegetable index and the livestock index across Ethiopian zones showed spatial variations of 0.57 and 0.12, respectively. In the selected spatial Durbin model, the deviance value was very small, indicating that the model fit the data well.

**Conclusion:**

The study found a high prevalence and significant spatial variation in both HRFB of birth and stunting across the Ethiopian zones. The spatial distribution of both HRFB of birth and stunting were found to be significantly clustered in the administrative zones of Ethiopia. These results emphasize the need for targeted interventions to address HRFB and stunting, supporting Ethiopia in achieving its Sustainable Development Goals.

## Introduction

Birth significantly influences population dynamics and impacts socioeconomic, demographic, health, and environmental development (1). The process from fertilization to delivery is fraught with risks (2). The Sustainable Development Goals (SDGs) aim to reduce preventable the under-five mortality (U5M) rate to 25 deaths per 1,000 live births by 2030 (3). In line with these goals, Ethiopia and other sub-Saharan African (SSA) nations are also engaged in efforts to lower the U5M rate of children to at least 25 deaths per 1,000 births by 2030. Crucially, U5M in SSA is primarily caused by high-risk fertility behavior (HRFB) and nutritional issues (3, 4). Children free from any risk may still fall under the category of inevitable risk (such as first-order births to mothers aged between 18 and 34 years) or are in the high-risk, but avoidable, category. HRFB of birth occurs when the mother is younger than 18 years or older than 34 years at the time of childbirth, when a child is born after a short birth interval (<24 months), and when the mother has high parity (more than three children) (5, 6). A single HRFB occurs when a mother reports one of these high-risk fertility characteristics during childbirth. Multiple HRFBs involve a combination of two or more of these factors during for a single child (7, 8). The most common HRFBs in Ethiopia are high parity (70.6%), advancing maternal age (56.6%), and closely spaced births sequentially (46.5%) (1, 9–11).

HRFB contributes highly to chronic under-nutrition in children, perpetuating a cycle of stunting across generations. It adversely affects several birth outcomes, including chronic undernutrition. HRFBs are associated with preterm births, intrauterine growth restriction, neonatal mortality, low birth weight, hypertension, stillbirth, premature labor, anemia, gestational diabetes, and nutritional problems in children (12–14). These risks are exacerbated by factors like child marriage, cultural taboos against contraception, lower education, insufficient health infrastructure, and harmful sexual behaviors (2, 10, 13, 14).

Currently, 149 million children under five are stunted globally, with 37% of Ethiopian under-five children affected (8, 15). Stunting, a major public health concern, was linked with higher mortality and severe morbidity in children, contributing to 45% of fatalities in under-five children (16, 17). In 2025, the World Health Organization (WHO) proposed a 40% decline in the prevalence of stunting and Ethiopia’s government has committed to ending child undernutrition by 2030 through initiatives like the “Sekota” Declaration and school feeding programs (5). Despite a decline in stunting prevalence in Ethiopia (from 58% in 2000 to 37% in 2019), the stunting rates still remain high, necessitating further study and intervention (18).

The majority of child stunting problem is linked to high-risk reproductive practices in Ethiopia (9, 18). Moreover, hazardous fertility practices are strongly associated with child undernutrition and poor birth outcomes (19). The primary global integrated public health issue is child stunting, which poses a serious threat to public health in developing nations like Ethiopia (20).

In Ethiopia, despite the implementation of national programs, child nutrition levels remain a pressing issue until now. Previous studies have linked various aspects of HRFB of birth to detrimental effects on children’s health. However, a thorough national-level research on the geographical relationship between HRFB and stunting in Ethiopia is lacking on under-five children in Ethiopia. Understanding this relationship is crucial for creating focused measures to enhance children’s health. At the smallest administrative levels, such as zones and districts, health indicators such as nutrition and HRFB of birth offer vital information required to enhance citizens’ health and address local health issues in vulnerable geographic areas. The estimation of the association between nutrition and HRFB of birth in small areas is a crucial indicator of child health condition.

While some studies have focused on particular regions, a thorough national-level research on the spatial relationship between HRFB of birth and stunting still needs to be done. The focus of this study is to explore the geographical relationship between stunting in children under five and HRFB across all the Ethiopian administrative zones. By identifying key factors and assessing regional and zonal prevalence, the study seeks to provide insights for targeted interventions to improve maternal and child health. Health indicators at the lowest administrative levels, such as regions and zones, provide essential information for policymakers to address local health concerns and implement effective interventions. This comprehensive approach will help policymakers and health practitioners address these critical issues more effectively, ultimately contributing to better health outcomes for Ethiopian children.

## Methods and statistical analysis

### Data sources and study population

**Figure 1:**
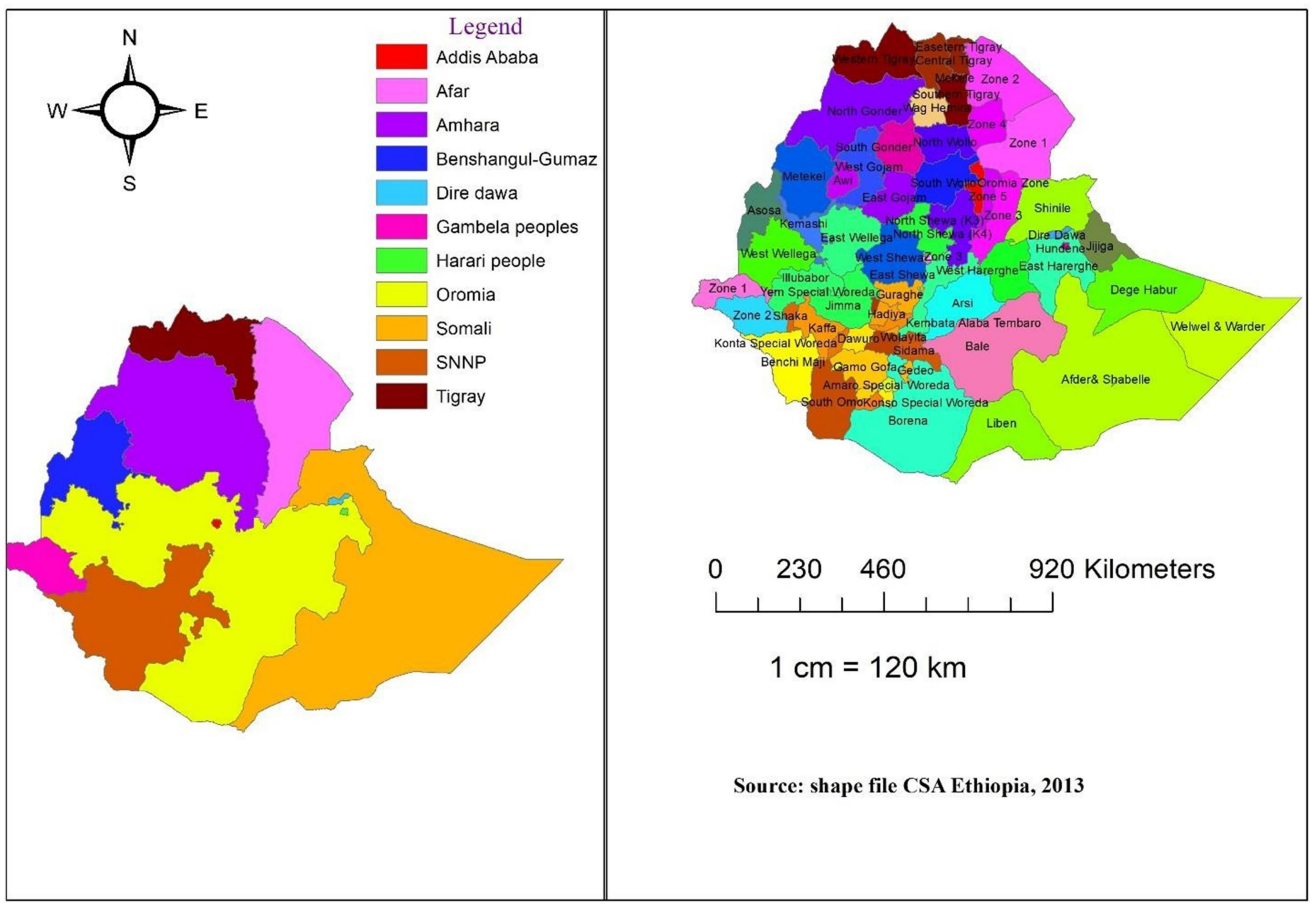
Structure of Ethiopian Regions (left) and Zones (right).

The data for this study were collected from 64 administrative zones in Ethiopia. Ethiopia is located in East Africa and covers a land area of 1.1 million km^2^. It is divided into 11 regional states, 64 administrative zones, 611 *weredas* (districts), and 15,000 *kebeles* (sub-districts) (Figure 1). Regions were divided into zones, zones into *weredas*, and *weredas* into kebeles, which are the smallest administrative units down to the household level (21). Ethiopia has implemented various economic development programs aimed at addressing key challenges such as stunting, poverty, hunger, illiteracy, and infant and maternal mortality. These programs include the Growth and Transformation Plan (GTP) and the Sustainable Development Goals (SDGs), which aim to improve the overall wellbeing of the population through targeted interventions in health, education, agriculture, and infrastructure development. Additionally, the country has also implemented initiatives such as the Productive Safety Net Program (PSNP), which provides support to vulnerable households to improve food security and resilience to shocks. These efforts reflect the government’s commitment to addressing the multifaceted challenges facing the country.

The Ethiopian Mini Demographic and Health Survey (EMDHS) was an important source of data for understanding the demographic and health characteristics of the Ethiopian population. Conducted by the Ethiopian Public Health Institute in collaboration with the Central Statistical Agency and the Federal Ministry of Health, the survey provides valuable insights into various aspects of health, including maternal and child health, nutrition, and access to healthcare services. The data collected from the EMDHS was crucial for informing policy decisions and designing targeted interventions to improve the health and wellbeing of the population. The EMDHS collects comprehensive data on men, women, and children across Ethiopia using a two-stage sampling process involving nine regional states and two city administrations. Individual sample weights (v005/1,000,000) were used in all analyses to account for over- and under-sampling.

Information from the Kids Record dataset, which comprises 5,753 records of information about mothers and their offspring, was also used. Women who had never married and married women who were sterilized were not included in the study sample. Additionally, the sample of women who did not give birth was not included in this study. After data cleaning, the final sample size included 5,074 children under five, with a weighted sample of 4,969 children. Mothers who had more than one child in the 5 years prior to the survey were asked about the most recent child during the mother’s interview for the survey. The dataset is easily accessible to the public and can be found at https://dhsprogram.com/data/.

### Data collection tools and procedures

All census enumeration areas (EAs) established for the 2019 Ethiopia Population and Housing Census (EPHC), conducted by the Central Statistical Agency (CSA), comprise the sample frame for the 2019 EMDHS. The census frame contains a detailed list of the 149,093 EAs that were created for the 2019 EPHC. A region classified as an EA normally has 131 households. The location of the EA, housing type (rural or urban), and the anticipated number of residential households are all included in the sampling frame. In order to provide estimates of the key indicators for the entire nation, separately for urban and rural areas, for each of the nine regions, and for the two administrative cities, the sample for the 2019 EMDHS was created. Utilizing a stratified, two-phase cluster sampling technique, the nationally representative sample was chosen. In the first phase, 305 EAs were selected with probabilities commensurate with the size of the EAs (93 in urban areas and 212 in rural areas) based on the 2019 EPHC frame. Using an equal probability systematic selection method, a fixed number of 30 households per cluster were chosen from the newly generated household list in the second stage of selection. The sampling units used for the initial sampling phase were called EAs. Households make up the second sampling stage in certain EAs. Finally, this study included a weighted sample of 4,969 children under the age of five (5).

### Study variables

The dependent and independent variables used in this study were depicted in the following sections.

### Response variables

The HRFB at birth and the stunting status of children (SSC) under five were the response variables in this study. Based on the following factors, HRFB of birth was developed from four components: (i) birth order greater than three; (ii) maternal age higher than 34 years; (iii) birth interval less than 24 months; and (iv) maternal age lower than 18 years. Children were considered to have experienced HRFB if any one of these criteria were met, resulting in a dummy variable with a value of 1 if HRFB was present and 0 otherwise (5, 10, 17, 19). The second outcome variable was the SSC under five, defined as a height-for-age Z-score below −2 SD (standard deviations) from the median of the reference population (15, 17). Since stunting was more common than wasting and underweight among Ethiopian children, it was chosen as the preferred anthropometric measure over the other ones. In Ethiopia, stunting remains a major concern, with high prevalence rates particularly among children under five other than anthropometric measure. Addressing stunting is crucial for improving child health outcomes and reducing child mortality rates in the country.

### Explanatory variables

Based on global and local literature reviews, potential independent variables that could be considered include: spatial disparities, geographical covariates, nutrition, healthcare access, socio-economic status, maternal health, environmental factors, and education. These are important factors that affect HFRB of birth and stunting collectively. This study considered predictors most related to the response variables of HFRB of birth and stunting together in a recent study. Geographical covariates for the spatial model were sourced and managed from geographical data set of EMDHS (1, 6, 10, 13, 17, 19, 22, 23).

### Statistical methodology

#### Spatial analysis

Spatial data refers to information about geographical locations, and spatial data analysis involves evaluating this data while considering spatial locations. Nearby attribute values are more statistically dependent than distant attribute values, which is a fundamental characteristic of spatial statistical models, a concept known as the first law of geography (24). This spatial dependence occurs when values at one location depend on neighboring observations, leading to unique analytical approaches. Proximity between zones impacts relationships through spatial autocorrelation, influencing spatial prediction methods based on regionalized variable theory (25).

### Spatial weight matrix

The spatial weights matrix W was n×n positive and symmetric matrix, as in Equation 3. The element of this matrix is $W_{ij}$ at location i,j for n locations. It plays a crucial role in describing the neighboring or spatial structures among locations. In the matrix, each row and column correspond to a specific unit area. These weights are often referred to as a neighboring function. By convention, the self-neighbor relation is excluded, so that the diagonal elements of W are zero$,W_{ij}=0$. More formally, $W_{ij}=1$ when i and j are neighbors, and $W_{ij}=0$ otherwise (26). That is given in Equation 1 as:


(1) $$W_{ij}=\{\begin{array}{c} 1\;\text{if}\;\text{zones}\;\left(\text{sites}\right)\;i\;\text{and}\;j\;\text{are}\;\text{connected}\\ 0\;\text{if}\;\text{zones}\;\left(\text{sites}\right)\;i\;\text{and}\;j\;\text{are}\;\text{not}\;\text{connected}\; \end{array}$$

To simplify interpretation, the weights matrix is commonly standardized by ensuring that the total sum of each row’s elements equals one. The elements of a row-standardized weights matrix thus equal. This is given in Equation 2 as:


(2) $$W_{ij}^{s}=\frac{W_{ij}}{\sum_{j=1}^{N}W_{ij}}$$

This makes sure that every weight is in the range of 0 to 1 and makes it easier to understand how to use the weights matrix to perform operations by averaging nearby values (27). If we assume that there are N spatial objects (regions or zones), then W be a square matrix of dimension NXN, which is given in Equation 3 as:


(3) $$W=\left[\begin{array}{c} \begin{array}{ccc} w_{11}\;w_{12}\;w_{13} & \cdots & w_{1N}\\ w_{21}\;w_{22}\;w_{23} & \ldots & w_{2N}\\ w_{31}\;w_{32}\;w_{33} & \cdots & w_{3N} \end{array}\\ \;\vdots \;\vdots \;\vdots \;\ddots \;\vdots \;\\ w_{N1}\;w_{N2}\;w_{N3}\;\ldots \;w_{NN} \end{array}\right]$$

The total numbers in a row *i* represent the total number of neighbors owned by location *i*. Weights can be defined in a variety of ways, including contiguity, distance, and other weights (26, 28–30). In this study, the use of a queen contiguity matrix to generate a neighborhood list based on edges and corners indicates that each location’s neighbors are defined by both shared borders (edges) and shared corners. This approach considers locations that are adjacent to each other either through a common boundary or through a point of contact at a corner. By employing a queen contiguity matrix, the analysis takes into account a broader definition of spatial relationships compared to other types of contiguity weights, such as rook contiguity (which only considers shared borders) or bishop contiguity (which only considers diagonal neighbors). The use of queen contiguity allows for a more comprehensive assessment of spatial connections between locations, capturing both direct and diagonal relationships. This choice of contiguity weights can influence the results of spatial analysis, as it determines which locations are considered neighbors and how their proximity is measured. By using a queen contiguity matrix, the study aims to capture a more inclusive set of spatial relationships among locations, which can provide valuable insights into patterns of spatial dependence and autocorrelation in the data. The element of spatial weight $W_{ij}=1$ shows that the location i and j are sharing a common edge or common vertex. Otherwise, $W_{ij}=0$ shows that the location i and j do not share a common edge and/or a corner each other (30).

Since in the case of this study we have only 64 administrative zones across the country, the corresponding weighted matrix is given in Equation 4 as:


(4) $$W=\left[\begin{array}{c} \begin{array}{ccc} 0\;w_{12}\;w_{13} & \cdots & w_{164}\\ w_{21}\;\;0\;w_{23} & \ldots & w_{264}\\ w_{31}\;w_{32}\;0\; & \cdots & w_{364} \end{array}\\ \;\vdots \;\vdots \;\vdots \;\ddots \;\vdots \;\\ w_{641}\;\;w_{642}\;w_{643}\;\ldots \;0 \end{array}\;\right]$$

We assume that the diagonal elements ($W_{11}$,$W_{22},\ldots ,W_{64}$) of this “spatial neighbor” matrix are set to zero. Because regions or zones are not neighbors to themselves. If two zones have a common border (on any side), they are considered spatial neighbors. In this instance, a border between two areas is deemed to be closer if it is longer than a predetermined “threshold distance.” Bishop’s point is where two spatial regions converge (30).

**Figure 2:**
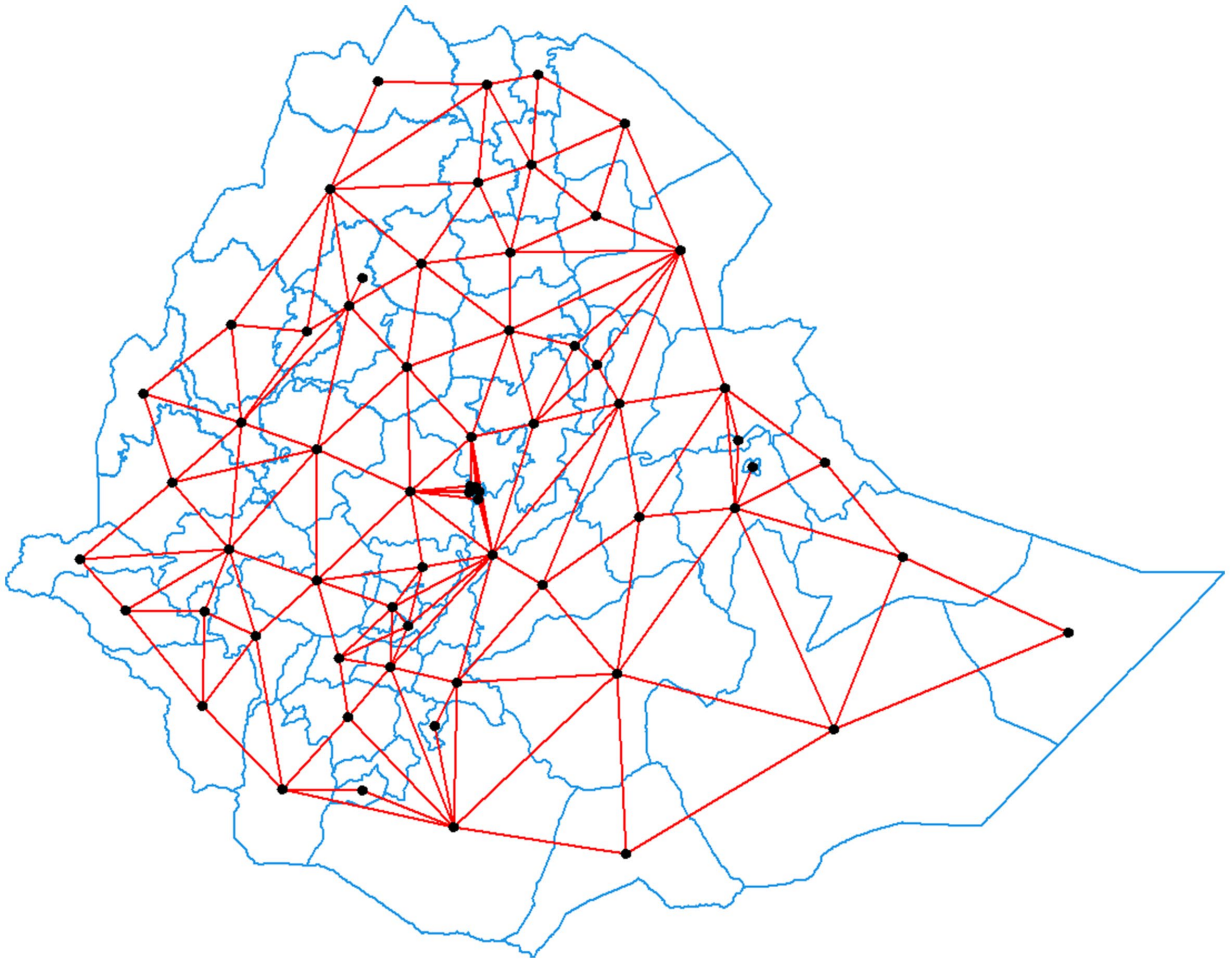
Queen contiguity and number of neighbors for each zone in Ethiopia. (Source: Survey data result 2019, Arc GIS).

This is similar to two graph elements intersecting at a vertex. Bishop contiguity occurs when two regions share a common border that is less than the threshold distance, whereas queen’s/king’s instance occurs when cells sharing a common edge or vertex are deemed contiguous (applicable for this study). Having number of 64 zones, the number of non-zero links is 372 with percentage non--zero weights of 7.175926, and average number of links of 5.166667 (Figure 2).

### Spatial autocorrelation

Spatial autocorrelation refers to the degree to which the values of a variable in a geographic area are correlated with the values of the same variable in neighboring areas. The spatial auto-covariance measures the relationship between nearby values of a variable, with the definition of “nearby” being determined by a $W_{ij}$(weighted matrix), which specifies the spatial relationships between locations. This measure quantifies how similar or dissimilar the values of a variable are at different locations, taking into account their spatial proximity. To produce a more useful spatial autocorrelation statistic, Moran’s I is commonly used. Moran’s I standardizes the spatial auto-covariance, providing a single value that indicates the overall level of spatial autocorrelation in the dataset. It allows for the assessment of whether similar values tend to be clustered together (positive spatial autocorrelation) or dispersed apart (negative spatial autocorrelation) across the geographic area under study. Moran’s I is widely employed in spatial analysis to understand patterns of spatial dependence and to identify clusters or spatial outliers in the data (26).

Global measures of spatial correlation are used to summarize the overall spatial pattern of a variable across an entire geographic area. These measures provide a single value that characterizes the degree of spatial correlation in the entire dataset, capturing the extent to which similar values tend to be clustered or dispersed across the entire study area.

One commonly used global measure of spatial correlation is Moran’s I, which quantifies the overall spatial autocorrelation in the dataset. Moran’s I takes into account the values of the variable at all locations and provides a single value that indicates the strength and direction of spatial correlation across the entire geographic area. A positive Moran’s I value suggests clustering of similar values, while a negative value indicates dispersion. This statistic is, which is given in Equation 5 as:


(5) $$\begin{array}{c} I=\left(\frac{n}{\sum \sum w_{ij}}\right)\;\frac{\sum \sum w_{ij}\left(x_{i}-\bar{x}\right)\left(x_{j}-\bar{x}\right)}{\sum {\left(x_{i}-\bar{x}\right)}^{2}}\\ =\left(\frac{n}{s_{0}}\right)\frac{\sum \sum wij\left(x_{i}-\bar{x}\right)\left(x_{j}-\bar{x}\right)}{\sum {\left(x_{i}-\bar{x}\right)}^{2}} \end{array}$$

where.

$W_{ij}$ are the elements of the spatial weighted matrix locations *i* and *j*,

$x_{ij}$ is the attribute value at the location of concern (location *i*) or (location *j*), and.

$\bar{x}$ is the average value of the attribute at the location.

The value of Moran’s I varies in the interval [−1, 1]. Interpreting this value is comparable to understanding correlation coefficients. Moran’s I will have a positive value if the values of the surrounding regions tend to be similar, and a negative value if the values of the neighboring regions tend to be different (31).

### Spatial interpolation and kriging interpolation

Spatial interpolation is a procedure for estimating values of a variable at non--sampled locations based on sampled location. Spatial Interpolation is part of a certain area can be predicted using observed data by a method called interpolation. Ordinary Kriging interpolation will be used to estimate the prevalence of HRFB of birth and (stunting statues of children (SSC) at non-sampled locations in Ethiopia. Ordinary Kriging is one of the most commonly used interpolation techniques and hence it is the best linear unbiased estimator (BLUE). The degree of proximity to the location being estimated, the quantity of data points in the immediate neighborhood at nearby data points, and the covariance function or semi-variogram of the data all have an impact on kriging. In this study, the use of SSC and HRFB of birth among children under five in unsampled regions of the nation was predicted using the spatial interpolation technique. Using the Ordinary Kriging Gaussian interpolation method, measurement errors were eliminated and forecast uncertainty was decreased (32).

### Spatial regression models

Numerous motivations for why spatial regression model relationships might arise was available than classical approach (non-spatial). Spatial regression models include time-dependence variable vector at time t, omitted variables, spatial heterogeneity, externalities, model uncertainty, and the interpreting parameter is also included (33). The zones served as the spatial unit of analysis for our investigation. The spatial location of data collected based on spatial specifications is not independent of its spatial location over time, a fact that is not taken into account by the traditional linear regression models estimated by ordinary least squares methods. The estimated values will not be impartial if the model ignores the spatial effects (11, 17, 19). Three different types of interaction effects can be identified in a spatial regression model: interaction effects among the error terms (*ε*), exogenous interaction effects among the independent variables (*X*), and endogenous interaction effects among the dependent variable (*Y*). Spatial relationships can be modeled in a variety of ways depending on the relationship between the dependent variable and the explanatory variables and error term. The most common approach in spatial analyses is to start from nested to specific, which is applicable also for this study. For all spatial model notation, the *n* × 1 vector **y** represents observations on the dependent variable, x represents an *n* × 1 vector of observations on a non-constant explanatory variable, *ε* represent various types of *n* × 1 disturbance vectors, *ρ*, *β*, *θ*, ʎ, and *δ* represent scalar parameters, and *W* is an *n* × *n* non-negative symmetric spatial weight matrix with zeros on the diagonal (28, 30, 31, 33, 34).

### General nested spatial model

#### General nested spatial model

The classical models did not include spatial analysis on the dependent variable, independent variables, and unobserved (error term) interaction effects with respect to the weight matrix. In order to account for the spatial effects of the outcome variable, independent variables, and error terms, we use seven spatial models. The outcome interaction effects between the explained variable, the covariate interaction effects between the explanatory variables, and the interaction effects between the error terms are the three categories of interactions found in the listed spatial model. General nesting spatial model (GNSM) is a compressive model with all types of the interaction effects and is the most general spatial models. Importantly, spatial autoregressive model (SAR), spatial Durbin mode (SDM), spatial Durbin error mode (SDEM), spatial lag model (SLX), spatial error mode (SEM), spatial autocorrelation model (SAC), and ordinary least square (OLS) can be modeled by considering different interactions. GNSM is a compressive model with all types of the interaction effects and is the most general spatial model.

This model is expressed in Equation 6 as:


(6) y=ρWy+Xβ+WXθ+u,u=λWu+ε

where y is a vector of observations on the dependent variable (nx1), X is a matrix of observations on the independent variables $\left(nxp\right)$ (contains both the individual and community level variables), Wy denotes the endogenous interaction effects among the dependent variable, WX the exogenous interaction effects among the independent variables, and Wu the interaction effects among the disturbance term of the different units. *ρ* is a spatial autoregressive coefficient and | ρ| < 1, θ is p × 1 independent covariates interaction effects, λ is spatial correlation effect of errors called spatial autocorrelation, β represents a p×1 vector of fixed but unknown parameters to be estimated, u is a vector of error terms (n×1) assumed to have autocorrelation.W is a n×n non-negative spatial weight matrix describing the spatial configuration or arrangement of the zones in the sample, *n* is the number of zones, and the error term *ε*, related to the spatial unit *s*, is identically and independently distributed with mean 0 and constant variance *εi*~ (0, *σ*2). This type of model contains all type of spatial interaction effect and breaks down to single spatial effect of variables.

Before selecting the model, which are best fitted to our data, we made a comparison based on their Akakian information criteria (AIC) and the least AIC were selected. Importantly SDM accounts only for spatial dependences among the independent covariates from nested model. This model incorporates the covariate spatial effect, y denotes the observation linked with a spatial unit, W is a non-negative n×n spatial weight matrix, and xi is a p×1 vector that represents values of *ρ* covariates recorded for the spatial unit and θ the independent covariates interaction effects. Furthermore, let zi be a q×1 vector that represents the values q of measured at spatial unit (26, 30, 33–36). The SDM model is given in Equation 7 as:


(7) y=ρWy+Xβ+WZθ+ε

## Results

### Descriptive statistics

**Figure 3:**
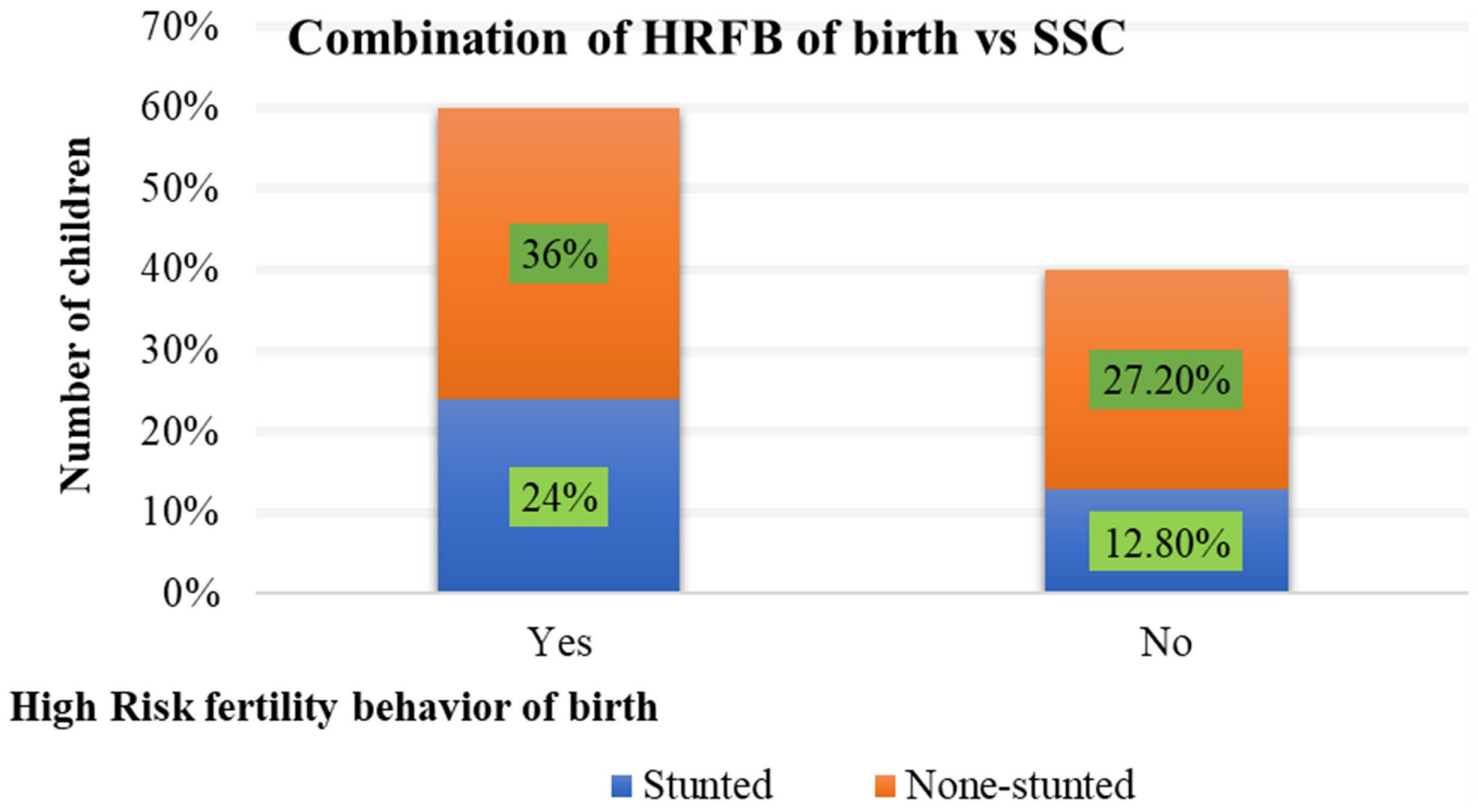
Percentage of association between HRFB of birth and Stunting.

The analysis included a weighted sample of 4,969 children under the age of five. Prior to model fitting, exploratory data analysis was conducted. As descriptive statistics, the relationship between each independent variable and the outcome variables was examined using both graphical analysis and chi-square test. The results showed that 60% of births have been identified as HRFB and 36.8% of children suffered from stunted growth. More importantly, 24% of under-five children were stunted due to HFRB (Figure 3).

### Spatial analysis

#### Spatial distribution of HRFB of birth and SSC

The explanatory variables considered in the spatial model analysis were evaluated in terms of proportion, with the zone as the unit of analysis. The spatial distribution of HRFB of birth and SSC across 64 Ethiopian zones were examined (Table 1).

**Table 1:** Descriptive statistics of the independent variables.

Variables	Minimum	1st Quartile	Median	Mean	3rd Quartile	Maximum	SD (CV %)	Moran’s I/Z values
HRFB of birth	0.15	0.54	0.61	0.59	0.69	0.83	0.16 (28.37)	0.28 (5.89)***
Severe stunting cases (SSC)	0.09	0.27	0.36	0.36	0.44	0.75	0.14 (37.88)	0.29 (6.05)***
Sex of household head: Women	0.0000	0.06	0.12	0.17	0.24	0.55	0.15 (91.4)	0.31 (0.63)***
Pregnancy counseling during pregnancy	0.0000	0.27	0.43	0.43	0.57	0.89	0.22 (51.15)	0.37 (7.65)***
Girl child	0.3	0.45	0.49	0.5	0.53	0.8	0.08 (15.34)	0.02 (0.7)
Health facility delivery	0.025	0.32	0.54	0.51	0.67	1.0000	0.25 (49.62)	1.07 (12.28)**
Residence in rural area	0.0000	0.74	0.86	0.79	1.0000	1.0000	0.29 (36.58)	0.49 (10.44)***
Cesarean section during deliveries	0.0000	0.0000	0.04	0.07	0.09	0.34	0.08 (127)	0.4 (8.47)***
Wealth Index	0.0000	0.16	0.27	0.35	0.48	1.0000	0.28 (80.18)	0.35 (7.29)***
Literacy	0.054	0.25	0.44	0.45	0.59	1.0000	0.23 (51.02)	0.4 (8.29)***
Antenatal care (ANC) visit	0.48	0.74	0.84	0.83	0.94	1.0000	0.13 (15.8)	0.38 (7.93)***
Postnatal check	0.0000	0.05	0.08	0.11	0.14	0.44	0.1 (90.57)	0.008 (0.28)
Twin children	0.0000	0.0000	0.0000	0.018	0.03	0.11	0.3 (147.6)	−0.03 (−0.31)
Age of children older than two years	0.4	0.55	0.58	0.58	0.61	0.69	0.06 (9.70)	−0.033 (0.35)
Number of under-five children in the household more than two	0.0000	0.03	0.12	0.14	0.2	0.6	0.14 (98.03)	0.36 (7.57)***
Media access	0.0000	0.2	0.28	0.35	0.43	1.0000	0.25 (71.78)	0.45 (9.38)***
Use contraceptive	0.0000	0.26	0.43	0.4	0.56	0.8	0.22 (55.03)	0.36 (7.46)***
Mean all population count	219	2,482	5,081	35,067	11,592	525,257	93,407 (266.4)	2.79 (35.66)**
Mean improved vegetation index	0.12	0.22	0.29	0.3	0.37	0.55	0.12 (38.64)	0.57 (1174)**
Mean maximum temperature	22.8	25.5	27.1	27.6	29.9	34.1	3.13 (24.08)	0.38 (7.82)***
Mean minimum temperature	9.36	11.29	13.03	13.87	16.27	21.64	3.34 (24.08)	0.37 (7.78)***
Mean under five population	21	437	814	3,054	1761	32,573	6,347 (207.8)	0.58 (13.1)***
Mean density of livestock	1.0000	83.30	175.30	189.5	251.90	632.50	133.1 (70.2)	0.12 (2.77)***

SD, Standard deviation; CV, Coefficient of variation; Moran’s I are significant at 10*, 5, and 1% = *, ** and ***.

The mean proportion of HRFB of birth and stunting at the zonal level in Ethiopia was observed to be 58 and 36%, respectively. Meanwhile, the proportion of households headed by women averaged 17% across the 64 zones. The first quartile of HRFB of birth and stunting was 0.54 and 0.27, respectively, while the third quartile was, respectively, 0.69 and 0.44, based on zonal aggregate prevalence. The median values for HRFB of birth and stunting were 0.61 and 0.36, respectively. Additionally, the mean proportion of pregnancy counseling was 43% in the Ethiopian zones. Out of the 64 Ethiopian zones included in this study, 51% of mothers made their deliveries in health facilities and 79% lived in rural areas.

Furthermore, the mean proportion of mothers from affluent households and those who were illiterate was 35 and 45%, respectively, across the 64 Ethiopian zones. The average zonal prevalence of media access and the use of contraceptives among Ethiopian mothers were 35 and 40%, respectively. The coefficients of variation for HRFB of birth and stunting in Ethiopian zones were presented as 0.27 and 0.28, respectively. These coefficients, along with those for significant independent variables, indicate wide variations among the zones in Ethiopia.

Significant autocorrelation, based on an adopted spatial weight matrix utilizing the queen adjacency criterion, indicates spatial autocorrelation between Ethiopian zones. Additionally, among the geographical variables, the mean maximum and minimum temperatures were observed to be 0.27 and 13.87, respectively. The spatial variation of the average vegetation index and the livestock index averaged 0.57 and 0.12, respectively, across Ethiopian zones based on this study (Table 1).

#### Spatial autocorrelation

**Figure 4:**
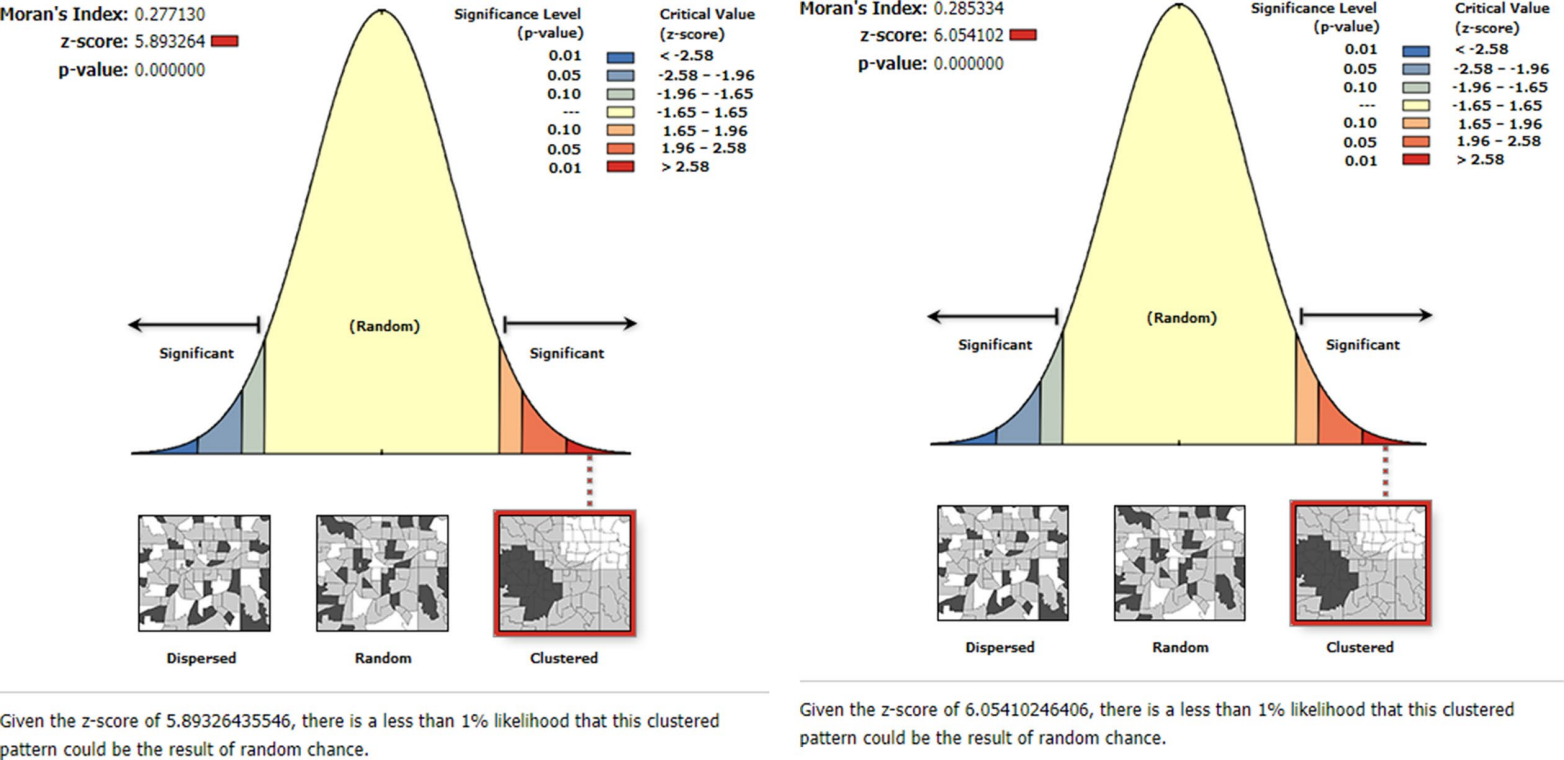
Spatial autocorrelation analysis for HRFB of birth (left) and stunting (right).

The estimated Global Moran’s I values for HRFB of birth and stunting were 0.277130 and 0.285334, respectively, which indicates that the spatial distribution of both HRFB of birth and stunting were significantly clustered across Ethiopian zones (*p*-value =0.05; Figure 4).

### Spatial distribution of HRFB of birth and stunting across Ethiopian zones

This study reveals that the proportion of children at high risk of birth and SSC varies across the 64 administrative zones included in the analysis. The zones with a high proportion of HRFB of birth, indicated by red color, are predominantly found in Somalia region zones (Afder, Welwel and Walder, Liben, and Jijiga zones), as well as zones two and four in the Afar region, Wag Hemira zone, South Gondar in the Amhara region, Hadiya, Kembata Tembaro, Alaba, and Sidama zones in the South Nation’s Nationalities and People’s Representatives (SNNPR) region, and Borena and North Shewa in Oromia region, in comparison to the rest of the zones.

Similarly, areas with a high proportion of SSC, indicated by a high prevalence of severe stunting cases, were observed in Tigray zones, Wag Hemira, North Gondar, South Gondar, North Wollo, Oromia special zone, and Awi zone in the Amhara region, East Harerghe, Borena, and North Shewa in the Oromia region, Konso Special Woreda and Guraghe in the SNNPR region, zone 3 in Afar region, and Asosa zone in the Benishangul-Gumuz region.

Conversely, zones with a low proportion of HRFB of birth, shown in green colour, are evident in all Addis Ababa zones and Konso Special Woreda in the SNNPR region. Similarly, for SSC, zones with a relatively low prevalence include all Addis Ababa zones, Dire Dawa administration, Welwel and Warder, Shaka, and Liben in the Somali region, and West Wellega zones in the Oromia region, compared to the rest of the zones in Ethiopia.

**Figure 5:**
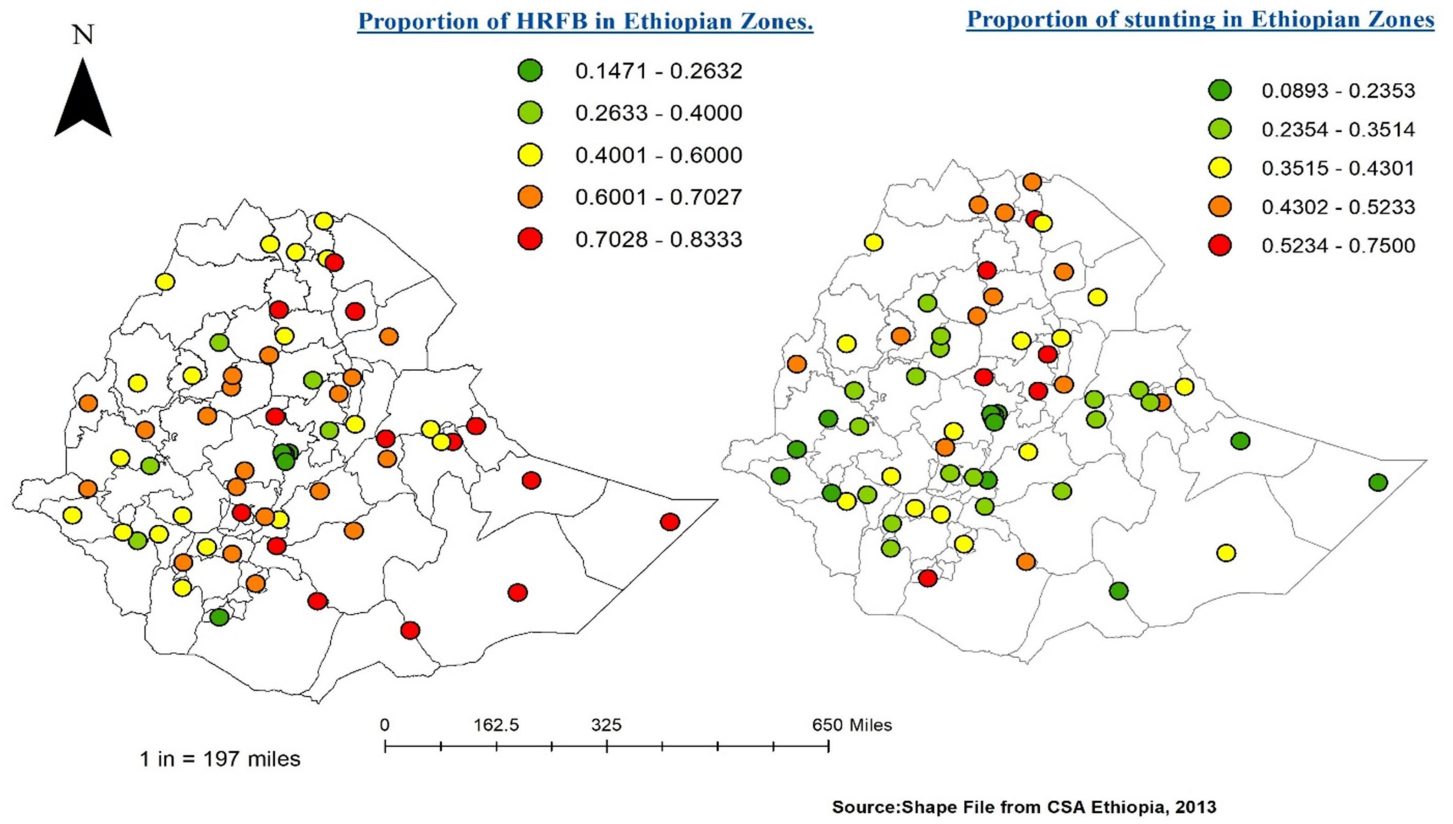
HRFB (left) and Stunting (right) across Ethiopian Zones.

Interestingly, certain areas such as Wag Hemira, zone 4, and zone 3 in Afar, South Gondar, Asosa, Oromia Special zone, East Harerghe, Borena, and North Shewa exhibit high proportions of both HRFB of birth and SSC simultaneously across the Ethiopian zones. Conversely, zones such as all Addis Ababa zones, West Wellega, and Bahir Dar special zone consistently display a low proportion of both HRFB of birth and SSC compared to other Ethiopian zones at same time (Figure 5).

### Hot-spot analysis of HRFB of birth and stunting status

**Figure 6:**
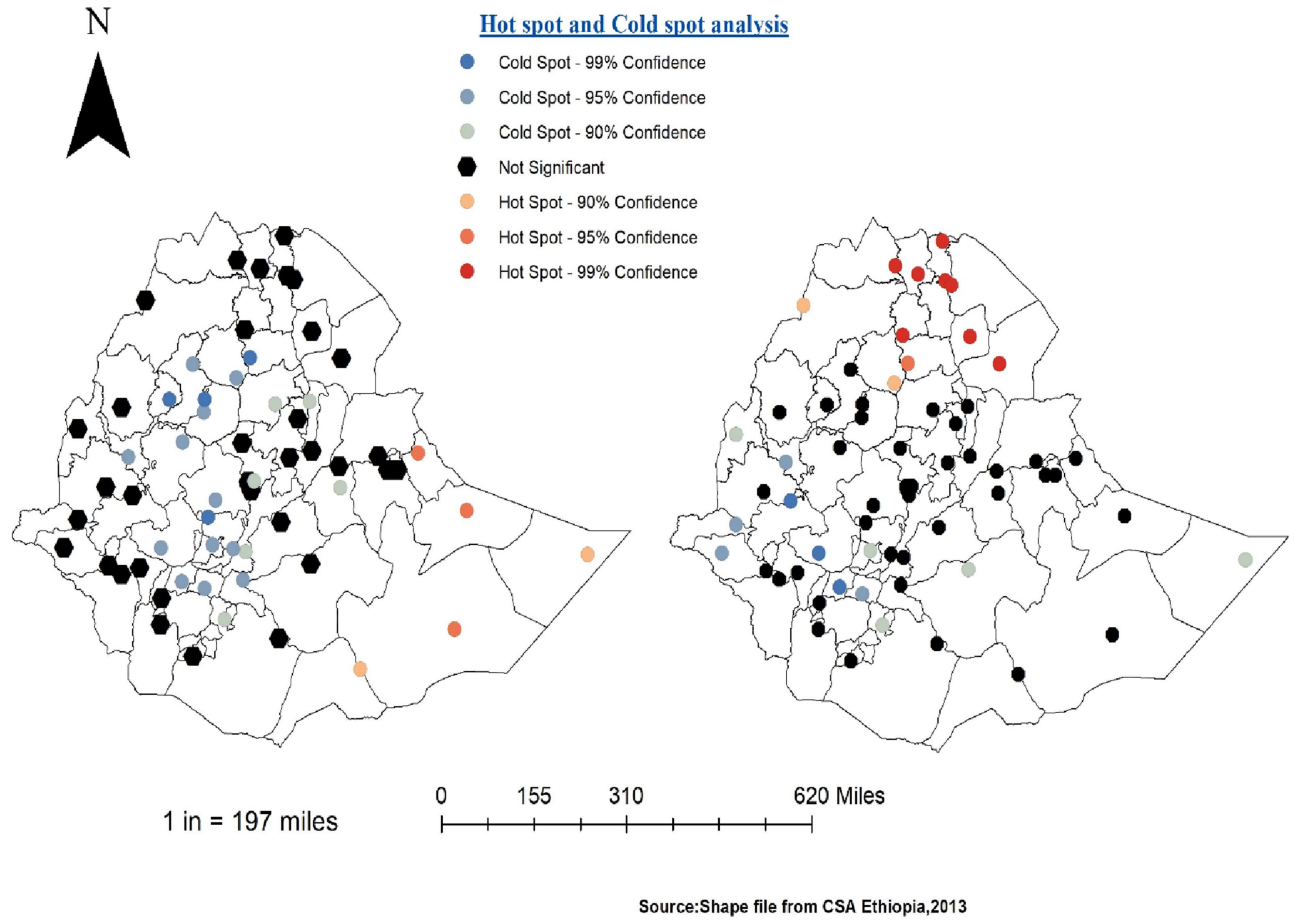
Hot-spot and cold-spot analysis of HRFB of birth (left) and SSC (right).

According to the local Getis-OrdGi* statistics, there are significant hot-spot and cold-spot areas for HRFB and SSC. The red colour indicates significant hot spot (high-risk) areas for HRFB of birth and stunting status, and the blue colour indicates the cold spots (low-risk) areas of HRFB and SSC. All Tigray region zones (Western, Eastern, Central and Southern), zone 1, zone 2, and zone 4 from Afar regions, Wag Hemira, North Gondar, South Gondar, and North Wollo zones from Amhara region were hot-spot areas for SSC, while the west part of the country was a cold-spot area for SSC. All Somalia zones (Afder zone, Welwel and Walder zone, Liben zone, and Jijiga zone) were hot-spots for HRFB of birth. In contrast, the Central, North-West, and South-West parts of the country were cold-spot areas for HRFB of birth. Both HRFB of birth and SSC have common share cold spots such as Jimma, Wolayita, Gedeo, Kemash, and Dawuro zones compared to other Ethiopian zones (Figure 6).

### Semi-variogram of HRFB of birth and stunting

**Figure 7:**
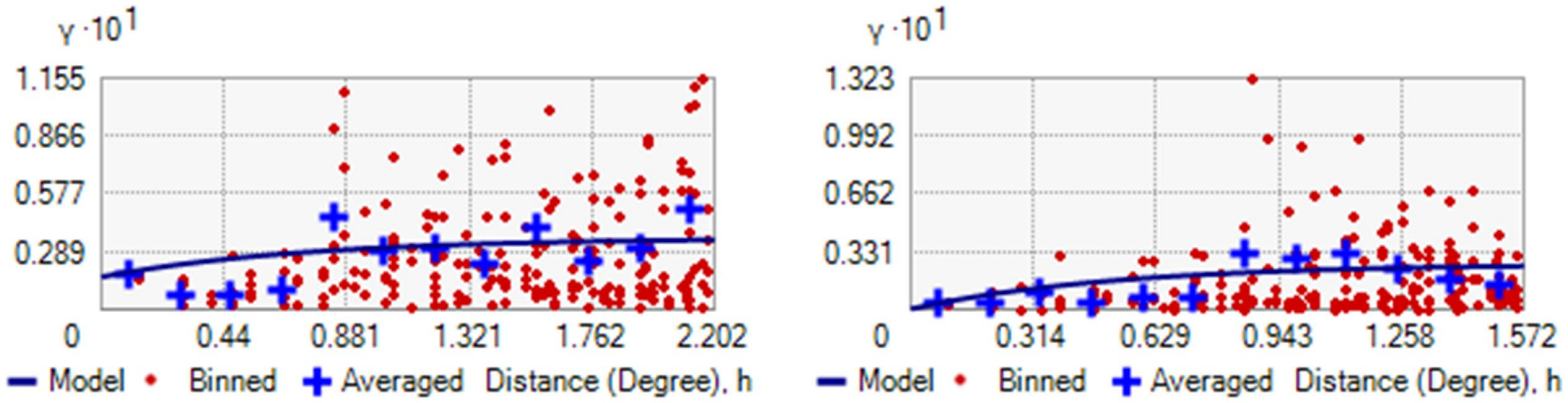
Semi-Variogram for HRFB of birth (left) and SSC (right).

Based on the mean square error (MSE), the exponential model was better (unbiased prediction) with a value of (0.1129) for HRFB and (0.1126) for stunting. Despite the small difference, the exponential model was preferred due to its closer proximity to zero. This model reflects a gradual decay in the relationship between two sample points (Figure 7).

The degree of spatial dependence is classified based on the nugget-to-sill ratio, which is expressed as a percentage. Ratios below 25% signify strong spatial dependence, ratios between 25 and 75% indicate moderate spatial dependence, and ratios above 75% suggest weak spatial dependence. In this study, the nugget-to-sill ratios for HRFB of birth and stunting were 0.0123 and 0.108, respectively. These ratios, reflecting spatial autocorrelation, were 1.23 and 10.8% for HRFB of birth and stunting, respectively. The nugget-to-sill ratio thus signifies the degree of spatial dependency. The results indicate a high spatial correlation of HRFB of birth and stunting across Ethiopian zones (see Table 2).

**Table 2:** Comparison of various semi-variogram models and their parameters.

Model	MSE for HRFB of birth	MSE for SSC
Circular	0.136	0.1132
Spherical	0.136	0.1131
Gaussian	0.165	0.1148
Exponential	0.134	0.1129
Nugget	0.00041	0.0027
Sill	0.03621	0.0250
Nugget-to-sill ratio (%)	0.0123 (1.23%)	0.108 (10.8%)

### Spatial model analysis

#### Model selection

The selection of the appropriate model was based on model comparison using evaluation criteria such as AIC, with the spatial Durbin model (SDM) having the smallest AIC selected for both HRFB of birth and SSC. All subsequent results were estimated and interpreted based on this SDM model (Table 3).

**Table 3:** Comparison of spatial models.

Model type									
		OLS	SLX	SAR	SEM	SDEM	SDM	SAC	GNSS
AIC	HRFB birth	−85.29	−98.8	−83.66	−102	−102	−129.2	−100.3	−100.3
	SSC	−121.1	−144.6	−120.6	−119.2	−119.2	−144.8	−121.6	−121.6

From direct effect, variables including pregnancy counseling, literacy, antenatal visit (ANC) visit, postnatal check, and the age of children older than 2 years showed significant associations with both HRFB of birth and SSC simultaneously. Notably, among geographical covariates, all population counts and maximum temperatures exhibited a positive relationship with both HFRB of birth and stunting. Conversely, enhanced vegetation index, minimum temperatures, under-five population, and density of livestock were negatively related to both high risk of birth and stunting Ethiopia at same time (Table 4).

**Table 4:** Parameter estimates of spatial regression model (SDM) for HRFB of Birth and SSC.

Parameters	Model estimates	
	HRFB of birth	SSC
	Estimate	Estimate
Intercept	−0.32657	−3.71838***
Sex of household head: women	0.19325*	−0.1861
Pregnancy counseling during pregnancy	−0.38362***	−0.45693***
Girl child	−0.53531***	−0.27503
Health facility delivery	−0.32657	−3.71838
Residence in rural area	−0.0741	0.35475***
Cesarean Section during deliveries	−0.19111	0.25529
Rich wealth Index	−0.06604**	−0.02116
Literacy	−0.14295**	−0.2576***
Antenatal (ANC) visit	−0.79858***	−0.71767***
Postnatal check	−0.33238**	−0.45252***
Twin children	−0.64826	−1.76977**
Age of children older than 2 years	0.84858***	−0.33337***
Number of under-five child in the household more than two	0.06676	0.06431
Media access	−0.02093	−0.08737
Use of contraceptives	−0.20572**	0.07165
Mean population count	0.1390	0.0450
Mean enhanced vegetation index	0.66915*	−0.07752**
Mean maximum temperature	0.03565**	0.04325**
Mean minimum temperature	−0.05017	−0.0512
Mean under-five population	0.00004	−0.00004
Mean density of the livestock	−0.0005	0.00018
Lag. Sex of household head: women	0.64566*	0.85208*
Lag. Pregnancy counseling during pregnancy	−0.57423	−1.19893**
Lag. Girl child	−1.50405***	−0.67312*
Lag. Health facility delivery	−0.97211***	0.83872**
Lag. Residence in rural area	−0.18618	1.00789***
Lag. Cesarean Section during deliveries	0.09347***	0.27621***
Lag. Rich wealth Index	−0.57324**	0.50931
Lag. Literacy	−0.14202	−0.82966*
Lag. ANC visit	−1.83328**	−0.82993
Lag. Postnatal check	−0.81299***	−0.20174
Lag. Twin children	−3.36277*	−2.71446*
Lag. Age of children older than 2 years	2.42025**	−0.12772
Lag. Number of under-five children in the household more than two	0.58652	0.08317
Lag. Media access	−0.00951	0.71784*
Lag. Use of contraceptives	−0.57423***	−1.19893
Lag. Mean population count	−0.03001	0.08041
Lag. Mean enhanced vegetation index	−0.87402*	1.56378***
Lag. Mean maximum temperature	0.01981	0.15769*
Lag. Mean minimum temperature	−0.07732	−0.16969***
Lag. Mean under-five population	0.00017***	−0.0001***
Lag. Mean density of the livestock	−0.00061**	−0.00012
AIC	−98.8	−144.6
Deviance	0.08785	0.09361
Bptest	45**	33**
ρ(rho)	−1.27	−0.3369
Jarque–Bera Test	1.8**	2.7**

Significant at * = (*a* = 0.05), ** = (*a* = 0.01) and *** = (*a* = 0.001).

From indirect effect, factors such as sex of the household head as women, girl child, health facility delivery, making Cesarean section, child twin, all population count, vegetation index, under-five population density of livestock, use of contraceptives, age of children, postnatal check, ANC visit, and wealth index, among others, showed a strong statistical association with high risk of birth and stunting in the 64 zones of Ethiopia simultaneously (Table 4).

A lower deviance value indicates a better fit of the model to the sample data. For HRFB of birth and SSC, the deviance values were 0.08785 and 0.09361, respectively, suggesting that the logistic regression model accurately describes the data. The Breusch–Pagan test was used to check for heteroskedasticity in the linear regression model, assuming that the error terms are normally distributed. This test evaluates whether the variance of the errors from the regression depends on the values of the independent variables (Table 4).

More importantly, the spatial coefficient (rho) in the SDM model was also statistically significant for high risk of birth and stunting. The coefficient of *ρ* was −1.274 and − 0.3369 for high risk of birth and stunting, respectively, hence statistically significant for the selected (SDM) model. This indicates that the explained variables of HRFB of birth and stunting, along with their interaction effect, were significantly influenced by neighboring zones. The Jarque–Bera test for residuals of HRFB of birth and SSC yielded results of [x-squared = 1.8, *p*-value (0.4)] and [x-squared = 2.7, *p*- value (0.3)], respectively, indicating that the data is normally distributed across Ethiopian zones for both HRFB of birth and SSC based on EMDHS 2019 (Table 4).

### Interpolation of the fitted model

According to the spatial distribution of fitted values for the risk of birth, the 10 areas with the highest risk include Borena, Liben, Hadiya, Wag Hemira, East Harerghe, Welwel and Warder, zone 4 of Afar, North Shewa (K4), Jijiga, Shinile, Afder and Shabelle, and Dege Habur. Conversely, the areas with the lowest risk for birth, after fitting the covariates, were Addis Ababa zones, Konso Special Woreda, Bahir Dar special zone, and South Wollo.

Similarly, for stunting, the highest incidence of SSC was observed in South Gondar, Konso Special Woreda, Wag Hemira, all Tigray zones, and North Shewa zone from Oromia among Ethiopia’s 64 zones using EMDHS 2019. The areas with the minimum SSC after fitting the covariates included all Addis Ababa zones, Shaka, Welwel, and Warder, based on the SDM of the zonal unit of analysis.

**Figure 8:**
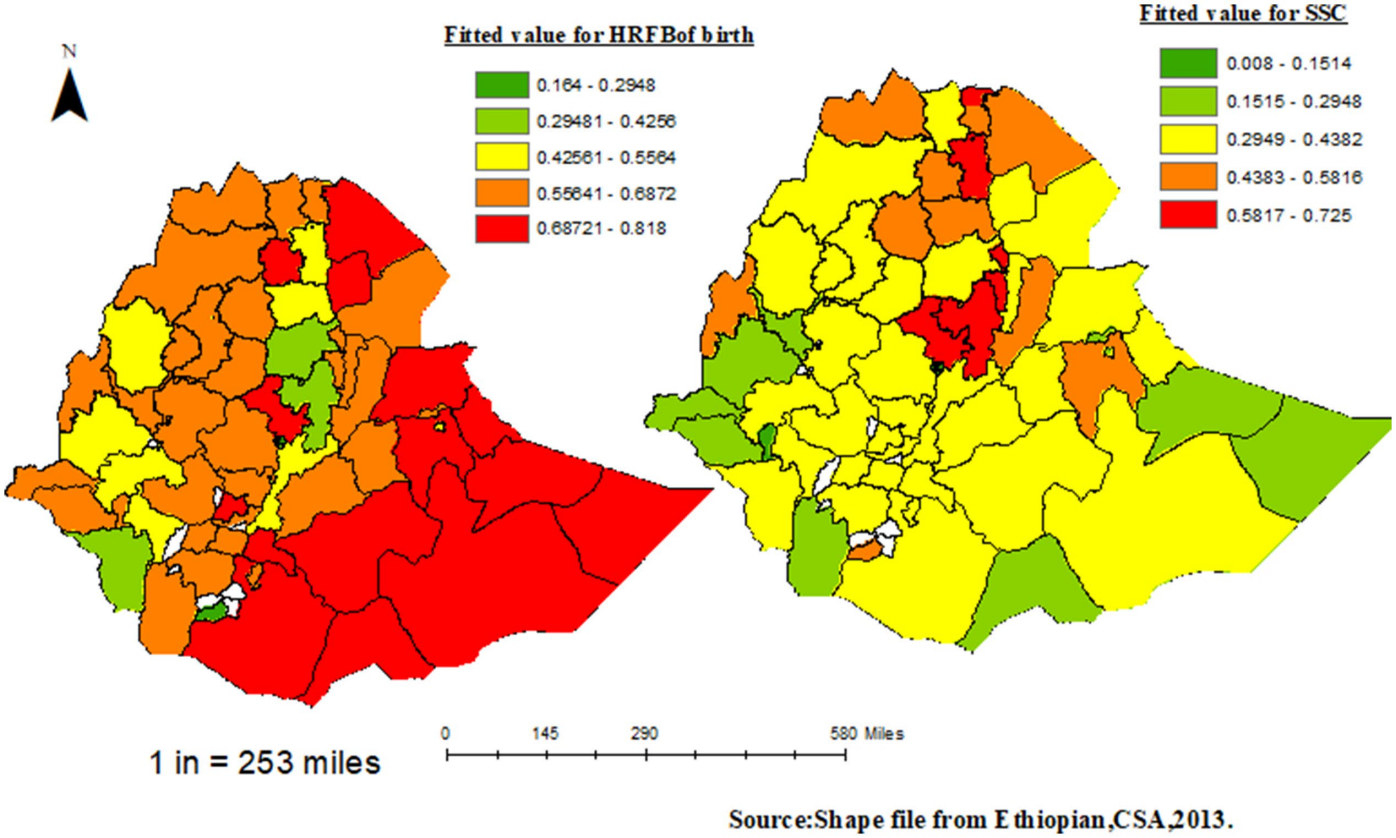
Distribution for fitted value of HRFB of birth (left) and SSC (right).

Notably, North Shewa, South Gondar, Eastern Tigray, and Wag Hemira zones simultaneously ranked high for both HRFB of birth and SSC. Conversely, all Addis Ababa zones and Bench Maji zones were found to have the minimum risk for both HRFB of birth and SSC compared to other zones based on EMDHS 2019 (Figure 8).

## Discussion

Our study offers an innovative approach of analysis that may be used to lower the probability of high-risk birth fertility behavior and chronic child undernutrition spatially (zone) across Ethiopia. Clinicians evaluating children with nutritional status issues may find our findings useful as a reference in other resource-constrained environments where chronic undernutrition and high-risk birth fertility behavior are prevalent.

This study used the spatial Durbin model (SDM) analysis across Ethiopian administrative zones to investigate the spatial association between stunting in under-five children and HRFB of birth. Various researchers have investigated the determinants of the spatial relationship between HRFB of birth and stunting in under-five children in Ethiopia and their findings support the determinants identified in our study (6, 9, 18, 37). To fully understand the elements influencing the spatial correlation between stunting in children under five and HRFB of birth, more statistical analysis is needed.

For the spatial zone aggregates data model SDM, several factors were significant for both HRFB of birth and stunting from the direct effect of the model. These factors include the sex of the household head (women), literacy, antenatal care (ANC) visits, postnatal checkups, age of children older than 2 years, under-five population, total population count, enhanced vegetation index, maximum and minimum temperatures, and livestock density. Similarly, from the indirect effects of the model, factors such as the sex of the household head (women), girl child, health facility delivery, Cesarean section, birth of twins, total population count, vegetation index, under-five population, livestock density, use of contraceptives, age of children, postnatal checkups, ANC visits, wealth index, minimum and maximum temperatures, media access, literacy, and pregnancy counseling have a strong spatial association with high risk of birth and stunting across the 64 zones of Ethiopia, which is consistent with a similar study (37, 38). This study’s findings are in line with previous research conducted in sub-Saharan Africa, Ethiopia, and the India (39–41).

The study, which examines the link between HRFB of birth and stunting, found that 1,177 children (24%) experienced stunting due to high-risk factors. These findings are consistent with research from Ethiopia, Bangladesh, and East Africa (10, 18, 22, 38). Moreover, the study revealed that HRFB of birth was observed in high proportions in the zones of Somalia, Afar, Amhara, Oromia, and SNNPR regions. In contrast, a low proportion of HRFB of birth was found in Addis Ababa, Tigray, Amhara, Harari, and Dire Dawa compared to other regions, similar to another study conducted in Ethiopia (9, 11, 19).

For stunting status clusters, highly clustered areas were in Tigray, Amhara, Afar, and Benshangul, while low cluster areas were in Addis Ababa, Gambela and Dire Dawa, Additionally, Wag Hemira, zone 3 from Afar, South Gondar, Asosa zone, Oromia Special zone, and North Shewa exhibited high rates of both HRFB of birth and SSC simultaneously. Conversely, all zones in Dire Dawa and Addis Ababa were consistently low in both HRFB of birth and SSC compared to other special zones (7, 18, 19). This might be because modern contraceptives are not widely used in Ethiopia’s Somali and Afar regions. Additionally, pastoralist communities frequently encounter restricted access to modern family planning services and health information due to their nomadic lifestyle and deep-rooted cultural and religious values. These results agree with those of other Ethiopian studies (16, 37).

Mothers who received pregnancy counseling during pregnancy reduced the risk of HRFB of birth and stunting, aligning with studies done in East Africa and Ethiopia (7, 9, 11). This is mainly because these mothers received a large amount of healthcare services from health stations. The study also identified the mother’s education level as a significant risk factor influencing the wellbeing of children in the country. Educated mothers are better equipped to provide proper nutrition for their children, as they possess greater knowledge, attitudes, and practices concerning nutrient-rich foods and maintaining a hygienic living environment. Children of less educated mothers are at a higher risk of HRFB at birth and stunting, which is consistent with findings from another study in sub-Saharan Africa and Ethiopia (11, 23, 41). This could be attributed to the fact that educated individuals have better knowledge and awareness about HRFB and a lower likelihood of experiencing early marriage.

Births from mothers who had difficulty accessing health services were more likely to experience HRFB at birth and stunting, consistent with previous research. This may be due to limited use of family planning and inadequate ANC and postnatal care among women who face barriers to accessing health services, leading to shorter birth intervals, births at older ages, and higher birth orders. Furthermore, the study discovered that mothers who received ANC during pregnancy and postnatal care were more likely to experience HRFBs than mothers who did not receive ANC. These findings are consistent with earlier research from Ethiopia, Bangladesh, India, and sub-Saharan Africa (10, 11, 13, 19). It was discovered that mothers who did not receive postnatal care (PNC) and ANC follow-ups for their recent pregnancies were more likely to exhibit risky reproductive behavior and to be stunted. Clinical examinations for the mother and the fetus are part of the ANC and PNC follow-ups, along with postnatal care counseling that includes family planning options to prolong the time between pregnancies. Consequently, HRFB and spatial stunting may be caused by the low use of ANC and PNC during pregnancy. Crucially, the study showed that, across Ethiopian zones, the likelihood of HRFB at birth and stunting increased with child age, which is consistent with research from East Africa, Ethiopia, and India (1, 13, 23).

This study also revealed that women with a poor wealth index were more likely to be at risk of HRFB than those with the richest wealth index, similar to studies in Nigeria, sub-Saharan Africa, and Ethiopia (11, 37, 42, 43). Mothers living in poverty often face challenges in affording basic necessities, which can increase their vulnerability to poor hygiene and sanitation practices, exposure to communicable diseases, and nutritional deficiencies. Previous research has linked mothers who have never used contraceptive methods with a higher occurrence of HRFB at birth compared to those who have used them, consistent with studies conducted in Nigeria, Kenya, and Ethiopia (1, 18, 20, 43–45). The goal of using contraceptives is to reduce the number of unplanned pregnancies and space out birth intervals, which can have serious health consequences for both the mother and the unborn child. Thus, by facilitating access to family planning services, essential postnatal services are vital in prolonging the time between pregnancies.

Births from households with a woman head increased the risk of HRFB of birth across Ethiopian zones, contrasting with some studies (12). The observed variation may be attributed to marriages of children practices, a high level of insufficient resources for family planning, and negative cultural misconceptions about women’s use of family planning. Births from rural households increased the risk of stunting, confirmed by studies done in sub-Saharan Africa, India, and Ethiopia (11, 18, 42, 46, 47). An enhanced vegetation index and maximum temperature increased the risk of HRFB of birth and stunting across Ethiopian administrative zones based on EMDHS 2019 data (39, 45, 48). This article introduces a robust heteroskedasticity-robust Breusch–Pagan test to evaluate the null hypothesis of zero cross-section (or contemporaneous) correlation in linear panel data models, without assuming independence among the cross-sections. Furthermore, the low deviation indicates little variation, and the Jarque–Bera test validates the data’s normal distribution (49). More generally our study highlights particular health issues or delivery gaps and makes recommendations for focused interventions that decision-makers could use. Think about talking about topics like health education, healthcare access, and resource allocation for affected each zone.

## Conclusion

The Ethiopian Mini Demographic and Health Survey (EMDHS) for 2019 provided the data used in this study. The results of this study showed that there was a considerable spatial variation in the prevalence of both HRFB of birth and stunting among the various zones in Ethiopia. Ethiopian administrative zones were found to have a significantly clustered spatial distribution of both stunting and HRFB of birth. We also found a variance in the rates of stunting and HRFB of birth in children under five at the zonal level.

Additionally, seven spatial regression models were employed to model the relationships between HRFB of birth, stunting, and various covariates among the administrative zones in Ethiopia. Importantly, we concluded that both HRFB of birth and stunting were higher in the Afar region, while Addis Ababa and Dire Dawa had the lowest rates of HRFB of birth and stunting compared to other regions. Furthermore, we found that Wag Hemira, zone 3 from Afar, South Gondar, Asosa, Oromia special zone, and North Shewa had high rates of both HRFB of birth and stunting, while all zones in Addis Ababa had consistently low rates. Our findings revealed that the SDM was better suited for capturing the interdependent nature of both HRFB at birth and stunting across Ethiopia’s administrative zones. These findings assist policymakers in revising programs to achieve Sustainable Development Goals (SDGs) for universal nutrition coverage among children under five in Ethiopia. In order to create successful interventions, supporting research projects that identify the causes of stunting and high-risk fertility behavior of birth is necessary. At the local, national, and international levels, laws that advance maternal health, child nutrition, and reproductive health services are needed.

### Limitations of the study

There are certain limitations and challenges in this study such as the lack of some variables and the presence of missing values in the Mini Demographic and Health Surveys (MDHS) as well as reporting and recall biases.

## Data Availability

Publicly available datasets were analyzed in this study. This data can be found at: https://dhsprogram.com/data/.
